# Valorization of Iron (II) Oxalate Dihydrate Coming from Pickling Processes through Thermal Conversion

**DOI:** 10.3390/ma17184630

**Published:** 2024-09-21

**Authors:** Emiliano Salucci, Antonio D’Angelo, Antonio Fabozzi, Osvalda Senneca, Francesco Bellucci, Rosa Francesca, Henrik Grénman, Henrik Saxen, Martino Di Serio, Vincenzo Russo

**Affiliations:** 1 Department of Chemical Science, University of Naples Federico II, Via Cintia 26, 80126 Napoli, Italy; emiliano.salucci@abo.fi (E.S.); antonio.dangelo@abo.fi (A.D.); diserio@unina.it (M.D.S.); 2 Department of Chemical Engineering, Åbo Akademi University, Henrikinkatu 2, 20500 Turku, Finland; henrik.grenman@abo.fi (H.G.); henrik.saxen@abo.fi (H.S.); 3 Institute of Sciences and Technologies for Sustainable Energy and Mobility of National Research Council, STEMS-CNR, P.le V. Tecchio 80, 80125 Napoli, Italy; antonio.fabozzi@stems.cnr.it (A.F.); osvalda.senneca@stems.cnr.it (O.S.); 4 CRdC Tecnologie Scarl, Via Nuova Agnano 11, 80125 Napoli, Italy; prof.bellucci@gmail.com; 5 Irpinia Zinco srl, Nucleo Industriale Calaggio, 83046 Lacedonia, Italy; rosa.francesca@gmail.com

**Keywords:** Iron (II) oxalate, thermal treatment, circular economy

## Abstract

The valorization of industrial byproducts is an emerging practice that aims to transform waste materials generated during production processes into valuable resources. In this work, a preliminary study was carried out on the thermal conversion of an industrial solid byproduct resulting from the pickling of metal surfaces, mainly containing iron (II) oxalate. In a fixed-bed reactor, the thermal conversion was investigated as a function of the operating temperature and overall time. The starting material and the products obtained after heat treatment were characterized in detail, using numerous qualitative and semi-quantitative techniques. The aim of this research was to determine the optimal operating conditions for the transformation of the industrial byproduct into a high-quality product. By varying the operating conditions, it was found that complete conversion of iron (II) oxalate to magnetite was achieved at high temperatures (i.e., 773 K and 873 K) after one hour of treatment. The resulting product had a low degree of crystallization, which increased slightly with an increasing reaction time at a temperature of 873 K, reaching a maximum of about 11%. The magnetite obtained can be used in the future as a starting material for chemical looping processes as a chemical/energy carrier for the production of hydrogen.

## 1. Introduction

In recent years, the environment and sustainability have become focus points for the scientific community and for industry. Specifically, in contemporary industrial processes, the valorization of secondary products and waste is becoming increasingly imperative. An example of residue valorization in an industrial process is pickling [1], which is a crucial step in metal surface treatment that is often used by the steelmaking industry to protect metal surfaces from impurities. Various formulations for performing pickling are available, but the traditional and most common one is a technique that involves hydrochloric or sulfuric acid. Hydrochloric acid is used at room temperature with a solution of 18% wt. Sulfuric acid is used at a temperature of 65 °C < *T* < 83 °C as a 0.2% wt. aqueous solution. The acid reacts with the metal foil and forms hydrogen at the interface. Sometimes, it can also penetrate deeply into the surface of the metal, causing cracks, which should naturally be avoided. In addition to the interaction with the oxides present on the surface, the aggressiveness of the pickling process can lead to the uncontrolled dissolution of the metal (i.e., oxidation state = 0). To prevent this, inhibitors such as oils, fats, or special organic compounds are added to the acidic solution to minimize and prevent the excessive removal of the metal from the lattice. In general, the amount of dissolved iron obtained during the process is between 0.30 ± 0.04% of the weight of the treated steel [2]. A new pickling agent, called GIPHOX and containing phosphoric acid, has recently been introduced to improve the process. The new acid mixture makes it possible to reduce the time required to remove the oxides by as much as 50% as compared to the traditional treatment with hydrochloric acid (diluted in 12–13% water) used at room temperature [3]. However, it should be noted that iron can dissolve in the HCl solution in concentrations up to 45–50 g/L and in green 78% wt phosphoric acid it can dissolve in concentrations that are even 10 times higher as compared to 33%–wt HCl. Due to the high cost of the supporting acids, the process is economically sustainable only if an efficient recovery process for the acid mixture is available. Oxalic acid (C_2_H_2_O_4_) is used to regenerate inorganic acid solutions [4]. It offers several distinct advantages as it is highly water soluble, has a strong chelating ability, and displays efficient dissolution and removal of metallic oxides, rust, scales, and other surface contaminants from metal substrates. Furthermore, oxalic acid exhibits a preferential affinity toward certain metal ions, enabling selective pickling treatments tailored for specific alloys and surface finishes. The efficiency of the oxalic, acid-based pickling processes is influenced by several factors, including concentration, temperature, pH, agitation, and contact time. The optimization of these parameters is essential for achieving desired pickling outcomes, while minimizing resource consumption and environmental impact. In addition to its technical merits, the utilization of oxalic acid in pickling processes necessitates a critical examination of its environmental implications. While oxalic acid itself is biodegradable and poses minimal ecological risks, the treatment and disposal of spent pickling solutions and associated wastewater require careful consideration. Efforts directed towards waste minimization, treatment, and resource recovery are imperative to mitigate environmental pollution and ensure sustainable practices within the metal finishing industry. The treatment of the abovementioned wastes with oxalic acid allows the production of iron (II) oxalate dihydrate Fe(C_2_O_4_·2H_2_O), which is also typically discarded as waste [5]. When the mixture of iron (II) oxalate and oxalic acid is separated from the acid mixture, it has the appearance of yellow mud and can be recovered, which is a great benefit of using organic acid. Two possible techniques can be used for recovering oxalic acid from iron oxalate: the chemical pathway and the thermal pathway. The chemical pathway uses hydrochloric or sulfuric acid, which involves the formation of salts, but it induces the high costs of acids and maintenance. Iron (II) oxalate dihydrate exhibits high thermal stability, a property that can be utilized in controlled decomposition processes. Through controlled heating, iron (II) oxalate dihydrate can be converted into various desirable products, including but not limited to iron oxides, carbonaceous materials, and metallic iron nanoparticles. The study of the thermal decomposition of iron oxalate is not particularly extensive [6] and often refers to materials prepared in situ in the laboratory, as in the study by Brown and Bevan [7]. In their study, the yellow solid was precipitated from ferrous sulfate solutions by adding oxalic acid to promote its precipitation. Thermogravimetric studies show that the weight loss of the sample occurred in a very narrow temperature range between 453 K and 493 K, with the weight loss being slightly above 50%. Using XRD techniques, the authors observed the presence of magnetite and metallic iron. Rao et al. [8] reported data on the decomposition in air of pure iron (II) oxalate and found that the sample completely decomposed into maghemite (γ-Fe_2_O_3_) in the presence of air at a temperature of 573 K. Angermann and Töpfer [9] contributed to the investigation of the thermal conversion process and confirmed that pure oxalate in the presence of air produces hematite as the main product and that magnetite can be obtained in the presence of a sufficiently low oxygen partial pressure. Depending on the operating temperature, it can lead to the formation of mixtures of different iron oxides including magnetite (Fe_3_O_4_), wüstite (FeO), and metallic iron. The reaction network is shown in Table 1 [10]:

The versatility of the products obtained from the thermal conversion of iron (II) oxalate dihydrate underscores the potential for multifaceted applications across different industries. Moreover, the valorization of iron (II) oxalate dihydrate through the thermal conversion pathways aligns with the principles of circular economy and sustainable development [11]. Furthermore, the diversity of the few results available in the literature on thermal processes applied to in situ samples makes it necessary to investigate and understand the decomposition phenomenon not only from an operational point of view but also in relation to the characterization of the solid itself. The presence of impurities could also change the structural and morphological properties of the industrial solids as compared to the pure samples, making it necessary to investigate specific thermal processes to valorize this byproduct. In this paper, we present a comprehensive investigation into the thermal conversion pathways of iron (II) oxalate dihydrate derived from pickling processes. The thermodynamics and kinetics governing the decomposition reactions were investigated. The chemical and structural properties of the materials were examined using X-ray diffraction. The results also give information about the potential uses of the materials. 

## 2. Materials and Methods

### 2.1. Materials

The yellow solid sample provided by Irpinia Zinco srl., Area Industriale Calaggio, Lacedonia 83046, AV, Italywas obtained by different filtration phases of acidic solutions from pickling processes, having been previously treated with oxalic acid and passed through a rotary filter and a vacuum belt. This process was necessary because the solid had a thixotropic behavior and therefore could be easily filtered with the usual filtration techniques. This product was finally ground to obtain a uniform particle size distribution (< 10 µm). The XRF analysis that was carried out to evaluate exclusively the metal content of the sample showed that the main component of the sample is iron (Fe); however, it also contained a small amount of zinc (Zn), phosphorus (P), chlorine (Cl), magnesium (Mg), sulfur (S), and traces of other components, as indicated in Table 2.

**Figure 1:**
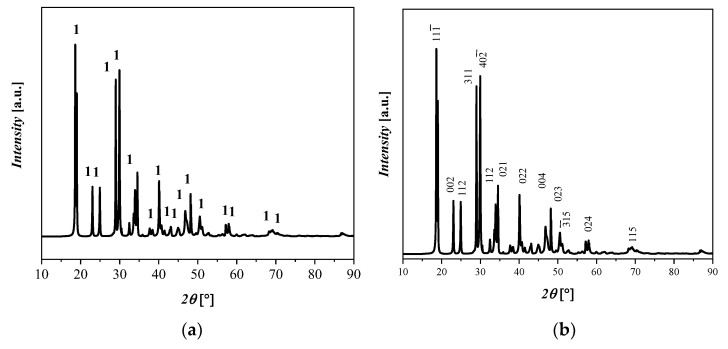
XRD diffractograms that display (**a**) reflections of Fe(C_2_O_4_) 2H_2_O and (**b**) the principle crystallographic plane.

An XRD pattern of the Fe(C_2_O_4_) 2H_2_O starting material (labeled here as 1), is shown in Figure 1, with its monoclinic crystalline structures with space group C 2/c and with the lattice parameters a = 11.79 Å, b = 5.53 Å, c = 9.61 Å, α = 90°, β = 126.4°, and γ = 90°. The RIR of Fe(C_2_O_4_) 2H_2_O is 90%, which is in relatively good agreement with the qualitative results of the XRF analyses, even if the Fe(C_2_O_4_) 2H_2_O content is underestimated with the RIR method because of the likely presence of different amorphous phases. The crystalline phase with ICDD codes is indicated: 1 = Fe(C_2_O_4_) 2H_2_O, 010910997.

### 2.2. Experimental Setup

**Figure 2:**
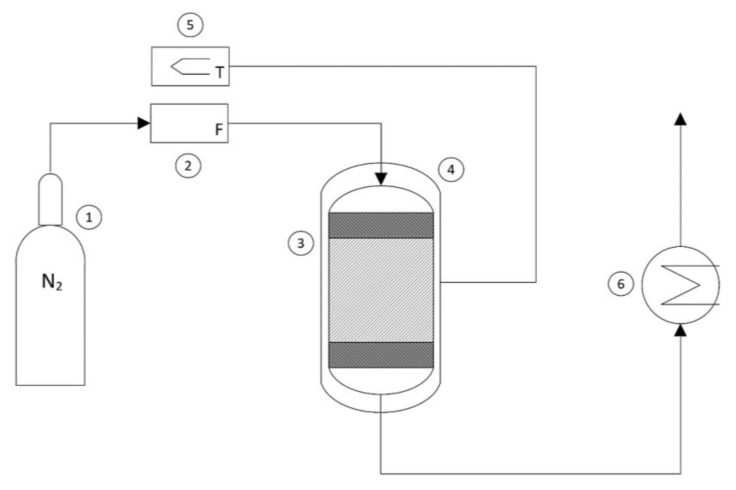
Schematic representation of the reactor system: (1) nitrogen bottle, (2) flowmeter, (3) reactor, (4) heating jacket, (5) thermoregulator, (6) heat exchanger.

The experimental thermal conversion tests were conducted in a continuous PBR reactor system in an inert nitrogen atmosphere, which was useful for the continuous removal of the gaseous effluents (H_2_O and CO_2_) generated during the decomposition of the oxalate. A schematic representation of the reactor system is shown in Figure 2.

The flow rate of nitrogen coming from a bottle (1) and fed into the reactor was regulated using a flowmeter (2) and set to 10 mL/min. The sample (approximately 100 mg) of iron (II) oxalate dihydrate was loaded into the reactor (3) and positioned between two layers of glass wool to prevent entrainment, thus forming a stable reactive solid bed. The cylindrical reactor had a diameter of 0.3 mm and a height of 10 cm. To evaluate the overall mass loss of the sample, the weight of the loaded reactor was measured before and after the thermal conversion process. By using an external heating jacket (4) connected to a thermoregulator (5), the experiments were conducted under different operating temperatures to assess the kinetics of the thermal decomposition process. Finally, for purely safety-related purposes, the hot gaseous effluents from the reactor were led into a glass system containing water at room temperature to quickly disperse excess energy (6). The thermal conversion experiments were conducted by varying two operating parameters: the temperature and the operating time. The experiments were conducted by varying the temperature with intervals of 100 degrees from 573 K to 873 K. Once the preset temperature was reached, thermal conversion was carried out for 30 min, 60 min, and 120 min.

### 2.3. Physicochemical Characterization

Thermogravimetric analyses (TGA/DSC) were conducted by coupling the Netzsch STA 449F1 (Selb, Germany) with a mass spectrometer to obtain a qualitative analysis of the gases released by the sample during the process. A qualitative analysis aimed at identifying the metal components of the starting material was performed using X-ray Fluorescence (XRF) with a Nexde-Rigaku instrument (The Woodlands, TX, USA). Subsequently, microstructural investigations of all the samples were examined via X-ray Diffraction (XRD) analysis, covering the 2θ range of 3–90°. This characterization was carried out by means of a Rigaku Miniflex 600 (The Woodlands, TX, USA) automated diffractometer equipped with a CuKα radiation source. Different phases were identified by using the PDF-5 + 2024 database from the ICDD International Centre for Diffraction Data^®^ in Newtown Square, PA, USA, along with the Rigaku Smart Lab II software v4.5.162.0. The determination of the Refraction Index Ratio (RIR), which is expressed in wt%, was executed by excluding the consideration of the amorphous phase [12,13].

## 3. Results

### 3.1. Thermal Conversion Analysis

**Figure 3:**
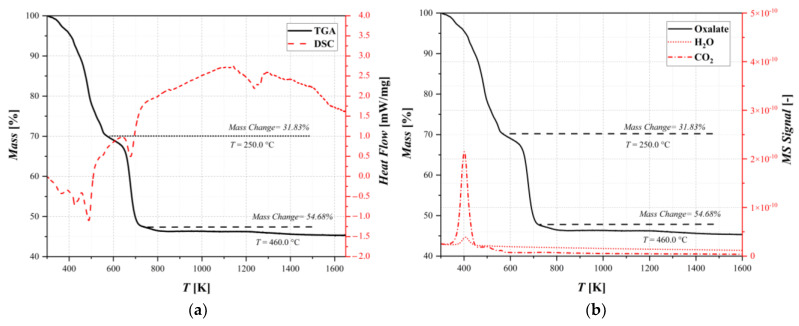
Thermal decomposition results for oxalate: (**a**) thermogravimetric analysis coupled with differential scanning calorimetry and (**b**) thermogravimetric analysis coupled with mass spectrometry.

Thermogravimetric/differential scanning calorimetry analysis (TGA/DSC) coupled with mass spectrum (MS) were performed to investigate the physical properties of the samples and evaluate the presence or amount of moisture and other compounds. The results are shown in Figure 3.

The differential thermal analysis is composed primarily of endothermic peaks. Figure 3a shows a weight loss of about 32% and a change in the decomposition rate at a temperature of T = 250 °C that could correspond to the dehydration–decomposition reaction. In fact, studying Figure 3b, this loss is significant, and it can be identified as the sum of the superficial water present in the sample and the CO_2_ related to the decomposition, according to the reaction scheme reported in Table 1. Subsequently, another rapid weight loss was observed at around 400 °C. This can be attributed to water crystalized in the lattice of the sample and a second release of carbon dioxide that formed after the thermal decomposition of the oxalate. Furthermore, the DSC results showed changes in the heat capacity, which correspond to phase changes in the sample. All of the changes in the weight loss rate show the point at which decomposition reactions start. It is also observed in the release of carbon dioxide and the subsequent phase changes through the heating ramp, indicating which of the decomposition reactions in the reaction pathway is the dominant one. The analysis shows that the decomposition is almost finished after a mass changes by about 54%, and it is confirmed in Section 3.2 thanks to the XRD analysis. However, this technique alone does not provide any further information on the discretization of thermal conversion pathways, taking into account the complex reaction network.

### 3.2. Thermochemical Kinetic Results

The experimental results show that when the temperature was increased from 300 °C to 500 °C, a phase transition was observed which was identified as the coexistence of Fe(C_2_O_4_) 2H_2_O and magnetite (Fe_3_O_4_), labeled as 2. The crystalline structure of Fe_3_O_4_ is cubic with space group Fd-3m and with the lattice parameters a = 8.31 Å, b = 8.31 Å, c = 8.31 Å, α = 90°, β = 90°, and γ = 90°. 

**Figure 4:**
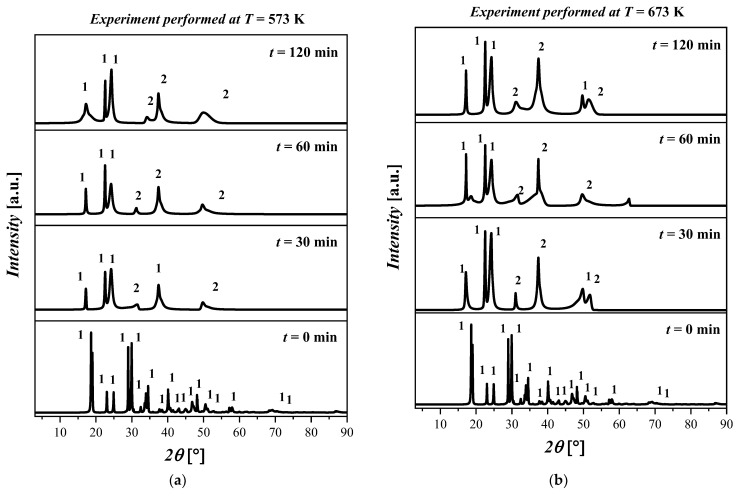

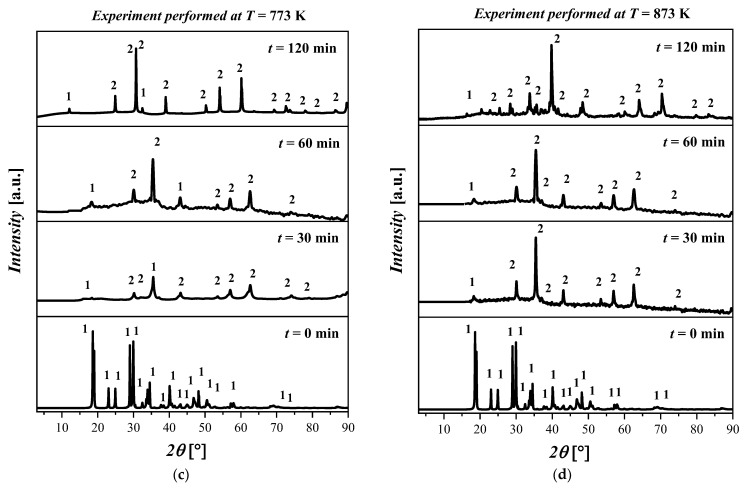
XRD pattern of the ((FeC_2_O_4_) 2H_2_O) starting material and after treatment at (**a**) *T* = 573 K for 30 min, 60 min, and 120 min; (**b**) *T* = 673 K for 30 min, 60 min, and 120 min; (**c**) *T* = 773 K for 30 min, 60 min, and 120 min; (**d**) *T* = 873 K for 30 min, 60 min, and 120 min. The crystalline phases with ICDD codes are indicated: 1 = ((FeC_2_O_4_) 2H_2_O, 010910997; and 2 = Fe_3_O_4_, 010861341.

In Figure 4a, no differences are observed for all the samples treated at *T* = 573 K. In particular, the XRD pattern shows the presence of both Fe(C_2_O_4_) 2H_2_O and Fe_3_O_4_ with a high amorphous content. In Figure 4b, the results for the thermal treatment at *T* = 673 K show a coexistence of Fe(C_2_O_4_) 2H_2_O and Fe_3_O_4_. In particular, by increasing the temperature, the presence of the amorphous phases was detected. Differences in the kinetics or morphology were not observed in the samples treated for 30 min, 60 min, and 120 min. The XRD patterns for all samples treated at *T* = 773 K are depicted in Figure 4c. The XRD patterns show that the increase in temperature resulted in the formation of Fe_3_O_4_. The increase in the reaction time from 30 min to 120 min resulted in an increase in the crystalline phase related to Fe_3_O_4_. The XRD pattern of the sample treated at this temperature for 120 min shows a clear crystalline phase attributable to Fe_3_O_4_; however, traces of Fe(C_2_O_4_) 2H_2_O were also detected. The XRD diffractograms of the samples treated at *T* = 873 K are displayed in Figure 4d. By inspecting the XRD patterns, it is possible to deduce that the crystallinity of the samples increased with the reaction time. In particular, the predominant crystalline phase attributable to the Fe_3_O_4_ is clearly observed after 120 min. The reflected rays can be explained by Bragg’s law:

*n* × *λ* = 2 × *d* × sin *θ* (1)

Bragg’s law mostly represents the crystalline portion of the samples, but when amorphous phases are present, some halo humps are featured. So, a degree of crystallinity (*DOC*) can be defined for the semicrystalline samples. In this work, the *DOC* with the integration method analysis has been evaluated. It was calculated from the XRD patterns by the following:

(2) $$\mathrm{DOC}=\frac{\mathrm{Area}\mathrm{of}\mathrm{crystalline}\mathrm{reflections}}{\mathrm{Area}\mathrm{of}\mathrm{crystalline}\mathrm{and}\mathrm{amorphous}\mathrm{reflections}}$$

The *DOC* was also determined for the starting material, and the results show a high crystallinity with a value of *DOC* = 98.92%. Subsequently, the same procedure was used to evaluate the *DOC* of the products after thermal conversion.

Nevertheless, additional analysis is desirable to understand the course of the reaction. In fact, the XRD evaluation gives information about the final products of the decomposition, but a more in-depth analysis should be made to identify the different steps of the reactions as well as the evolution of the *DOC* while the process occurs.

## 4. Discussion

**Figure 5:**
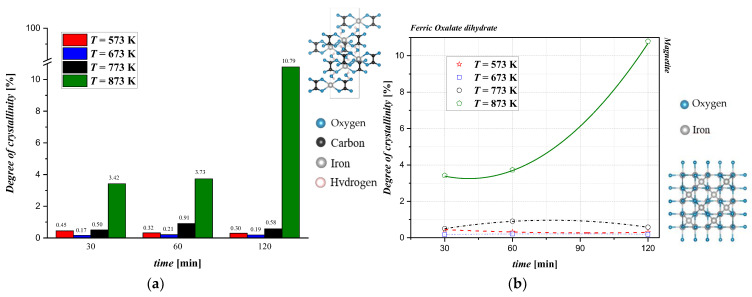
(**a**) Quantitative evaluation of the degree of crystallinity (*DOC*) after thermal conversion at different reaction times and temperatures and (**b**) qualitative evaluation of the degree of crystallinity after thermal conversion at different times and temperatures.

In this work, a preliminary study of the thermal conversion process of iron (II) oxalate was carried out to determine the best operating conditions for the valorization of this industrial byproduct. The comparison with the results from the literature, which mainly refer to the thermal conversion of pure samples, shows deviations in several aspects. Fundamentally, the TGA/DSC analyses performed by different authors show that the weight loss of the samples starts at 463 K, with a total mass loss of 50% in a temperature range significantly lower than that observed in the present work (i.e., 453 K to 518 K) [8]. Furthermore, depending on the oxygen partial pressure in the reaction environment, the final product can change from magnetite to the more oxidized form hematite [9]. In an inert environment, the formation of both magnetite and metallic iron was observed [7]. Hermanek et al. [10] reported that TGA showed a double phase of weight loss during the conversion of pure iron (II) oxalate (Sigma Aldrich), similar to the results reported in this article. In particular, between 453 K and 493 K, only the release of water (about 15% of the total weight) was observed, followed by a much longer standstill phase than for the industrial byproduct, where the weight remained almost unchanged until 643 K. By analyzing the samples at precise time intervals, the authors show that anhydrous oxalate converts into various chemical species in addition to magnetite, including iron carbide (*T* < 693 K), wüstite (*T* > 873 K), and iron. These species are not identified in the present work in experiments at different overall times, nor is the presence of carbon monoxide evident from the mass spectrometry results. The presence of CO is necessary to provide the necessary reducing reaction environment useful for the formation of wüstite from magnetite. The discrepancy with the characterization results obtained in this work inevitably means that the effects of impurities and solid morphology are considered crucial for determining optimal operating conditions and, in the future, decomposition kinetics. The differences identified between this work and the scientific literature also raise some questions regarding the techno-economic analysis of the thermal conversion process. The determination of the final chemical target species is crucial, because it is necessary to define the working condition in the presence of air or in an inert environment where high temperatures are reached. Further work is therefore required to evaluate the conversion kinetics (e.g., by assessing the composition of the off-gases exiting the tubular reactor) and to maximize the quality of the final product obtained. The relationship between the degree of crystallinity and the reaction time obtained from XRD at different temperatures is displayed in Figure 5. As previously described, oxalate is a crystalline material; however, the crystallinity decreases rapidly during thermal treatment. For samples treated at *T* < 873 K, it is not possible to obtain samples with a *DOC* above 3%. Specifically, at *T* = 573 K, the *DOC* monotonously decreases with the time of thermal conversion. It is reasonable to attribute this to the amorphous compounds, which are a mixture of magnetite and oxalate, which do not thermally decompose. At *T* = 673 K, the product has a low *DOC* value, which may be ascribed to an incomplete thermal conversion by iron (II) oxalate that is partially rearranged in magnetite. In this case, the trend is a bit different because a maximum *DOC* value of 0.21% was observed for *t* = 60 min. At *T* = 773 K, the crystallinity values differed for the different reaction times. A maximum *DOC* = 0.91% at t = 60 min was observed, which corresponds to a magnetite phase detected in the product analysis even if oxalate was still present. Finally, at *T* = 873 K, the highest *DOC* values were obtained. The *DOC* increased with the reaction time up to a value of 10.79%. This value corresponded to a phase in which almost the majority of the magnetite was present in a pattern with more definite and resolute reflections. In conclusion, comparing this value with the *DOC* of the starting material, it may be concluded that the samples underwent a rapid loss of crystallinity in the initial stages of the heat treatment. However, it is important to underline that the degree of crystallinity increased with increasing temperature and treatment times. Moreover, the increase in crystallinity can be attributed to the molecular mobility at elevated temperatures, facilitating the formation of crystalline domains. As the annealing progressed, the system became more ordered, and the crystallization was favored due to the complete conversion from oxalate to magnetite but also to the reduction in the amorphous fraction available.

## 5. Conclusions

The valorization of the yellow mud, composed of iron (II) oxalate (FeC_2_O_4_) and oxalic acid, is needed to optimize the pickling process. Despite the potential industrial interest, only a few results are reported in the literature on the kinetics of the mentioned reaction; investigation is surely needed to understand both the structural and morphological changes during the thermal processes. In this work, the starting material provided by Irpinia Zinco srl was characterized, and the thermal conversion was investigated, respectively, as follows:

- The diffraction patterns show that it was composed mainly of iron oxalate dehydrate. The TGA/DSC analyses display two weight losses that correspond to a release of CO_2_, suggesting the initial thermal conversion of the material and a release of water crystallized in the lattice of the sample.
- The thermal conversion shows that iron oxalate decomposes through a complex reaction mechanism involving several steps. The results, analyzed via the X-ray diffraction technique, indicate that a high temperature promotes the formation of magnetite over other oxides. At temperatures of *T* = 573 K and *T* = 673 K, the resulting product was a mix between the oxalate that was still present and the magnetite at time *t* = 120 min, with an amorphous background. At temperatures of 773 K and 873 K, the XRD pattern shows the formation of magnetite just after 60 min, which also had a rather high crystallinity.

Further investigations into the influences of temperature, heating rate, and time of reaction are needed to fully understand the kinetic reaction patterns, morphology, and phase evolutions. These will make it possible in the future to optimize the process and make the production more sustainable.

## Figures and Tables

**Table 1:** Reaction pathways of the thermal conversion of iron (II) oxalate dihydrate.

Steps	Reaction
1	FeC_2_O_4_ ·2H_2_O ⇒ FeC_2_O_4_ + 2H_2_O
2	3 FeC_2_O_4_ ⇔ Fe_3_O_4_ + 4CO + 2CO_2_
3	3 FeC_2_O_4_ + 2CO ⇔ Fe_3_C + 7 CO_2_
4	Fe_3_C ⇔ 3Fe + C
5	Fe_3_O_4_ + CO ⇔ 3 FeO + CO_2_
6	4FeO ⇔ Fe_3_O_4_ + Fe

**Table 2:** Elemental composition of the iron (II) oxalate dihydrate sample.

Elements	Area [%]
Fe	74.3
Zn	9.16
P	7.67
Cl	3.78
Mg	1.77
Ca	0.0707
V	0.0463
Sn	0.0395
La	0.0389
Cu	0.0348
S	1.11
Al	0.965
Si	0.349
Mn	0.305
Ni	0.180
Cr	0.135
Br	0.0106
Mo	0.0076
Ag	0.0073
Rb	0.0054
Y	0.0049
As	0.0041

## Data Availability

Data are contained within the article.

## Notation

Abbreviations	
DOC	Degree of crystallinity
DSC	Differential scanning calorimetry
MS	Mass spectrum detector
PDF	Powder diffraction file
RIR	Refraction Index Ratio
TGA	Thermogravimetric Analysis
XRD	X-ray Diffraction
XRF	X-ray Fluorescence
Symbols	
d	Grating constant
n	Diffraction order
T	Temperature
T	Time
λ	Wavelength
θ	Glancing angle
